# COVID-19-bezogene Selbststigmatisierung: Zusammenhang mit Vulnerabilität und Vertrauen in Institutionen

**DOI:** 10.1007/s00103-023-03742-z

**Published:** 2023-07-03

**Authors:** Nadine Reibling, Philipp Linden, Torsten Schneider

**Affiliations:** 1 Fachbereich Gesundheitswissenschaften, Hochschule Fulda, Fulda, Deutschland; 2 Seminar für Sozialwissenschaften, Universität Siegen, Adolf-Reichwein-Str. 2, 57068 Siegen, Deutschland

**Keywords:** COVID-19, Vulnerabilität, Stigma, Vertrauen, Geschlecht, COVID-19, Vulnerability, Stigma, Trust, Gender

## Abstract

**Hintergrund:**

Selbststigmatisierung kann im Rahmen von Infektionskrankheiten eine psychosoziale Belastung darstellen und die Bereitschaft, Infektionsschutzmaßnahmen einzuhalten, reduzieren. In dieser Studie wird erstmalig untersucht, wie COVID-19-bezogene Selbststigmatisierung in Deutschland in Gruppen mit unterschiedlicher sozialer und medizinischer Vulnerabilität ausgeprägt war.

**Methoden:**

Datengrundlage bildet eine webbasierte Befragung (CAWI: Computer Assisted Web Interview), die während der COVID-19-Pandemie im Winter 2020/2021 durchgeführt wurde. Die Quotenstichprobe (*N* = 2536) ist repräsentativ für die deutsche Erwachsenenbevölkerung hinsichtlich zentraler soziodemografischer Merkmale (Geschlecht, Alter, Bildung, Wohnort). Zur Operationalisierung der COVID-19-bezogenen Selbststigmatisierung wurde eine selbst entwickelte Skala verwendet. Außerdem wurden Daten zur medizinischen und sozialen Vulnerabilität sowie zum Vertrauen in Institutionen erhoben. Die Auswertung erfolgte mittels deskriptiver Statistiken und multipler OLS-Regressionen (engl. Ordinary Least Squares).

**Ergebnisse:**

Insgesamt liegt die durchschnittliche Selbststigmatisierung leicht über dem Skalenmittelwert. Während hinsichtlich sozial vulnerabler Gruppen nur Frauen eine erhöhte Selbststigmatisierung angeben, weisen Personen mit medizinischer Vulnerabilität (hohe Infektionswahrscheinlichkeit, schlechter Gesundheitszustand, Zugehörigkeit zur Risikogruppe) signifikant höhere Werte auf. Ein höheres Vertrauen in Institutionen ist mit stärkerer Selbststigmatisierung assoziiert.

**Diskussion:**

Stigmatisierung sollte in Pandemien regelmäßig monitort und für Kommunikationsmaßnahmen berücksichtigt werden. Es ist wichtig, auf weniger stigmatisierende Formulierungen zu achten und auf Risiken hinzuweisen, ohne Risikogruppen zu definieren.

**Zusatzmaterial online:**

Zusätzliche Informationen sind in der Online-Version dieses Artikels (10.1007/s00103-023-03742-z) enthalten.

## Abstract (en)

**Background:**

Self-stigmatization in the context of infectious diseases can be a psychosocial burden and reduce the cooperation with infection control measures. This study investigates for the first time the level of self-stigmatization among individuals with different social and medical vulnerabilities in Germany.

**Methods:**

Data are derived from an online survey (CAWI: Computer Assisted Web Interview) conducted during the COVID-19 pandemic in winter 2020/21. The quota sample (*N* = 2536) is representative for the key variables gender, age, education, and place of residence for the German adult population. For operationalizing COVID-19 related self-stigmatization, we developed a new scale. We also collected information on medical and social vulnerabilities as well as trust in institutions. Data analysis was based on descriptive statistics and multiple ordinary least squares (OLS) regression.

**Results:**

Overall, we find a level of self-stigmatization slightly over the scale mean. While socially vulnerable groups do not have higher levels of self-stigmatization – with the exception of women – individuals with medical vulnerabilities (higher infection risks, poor health status, risk group) show higher levels of self-stigma. Higher trust in institutions is associated with higher levels of self-stigmatization.

**Discussion:**

Stigmatization should be regularly monitored during pandemics and considered in communication measures. Thus, it is important to pay attention to less stigmatizing formulations and to point out risks without defining risk groups.

## Einleitung

Über zwei Jahre beeinträchtigte die COVID-19-Pandemie das gesellschaftliche Leben und führte nicht nur zu zahlreichen Todesfällen, sondern hatte viele weitere dramatische gesundheitliche und soziale Folgen. Eine weniger beachtete Konsequenz sind die Stigmatisierungsprozesse, die im Kontext von Pandemien auftreten [1]. Unter Stigmatisierung wird ein Prozess verstanden, bei dem Individuen oder Gruppen mit negativen Attributen belegt, hierdurch diskreditiert werden und ihnen die soziale Akzeptanz verweigert wird [2]. Vorhandene Studien zeigen auf, dass Stigmatisierungen im Kontext der COVID-19-Pandemie als Folge einer Infektion auftraten oder als Reaktion auf soziale Charakteristika, die in einer Gesellschaft mit einem erhöhten Infektionsrisiko in Verbindung gebracht werden (u. a. ethnische Zugehörigkeit, Tätigkeit im Gesundheitswesen; [3, 4]). Die weltweite Prävalenz, eine krankheitsbezogene Stigmatisierung bei einer COVID-19-Erkrankung zu erleben, wird dabei auf 34 % geschätzt [5].

Bereits seit dem Beginn der Pandemie riefen Wissenschaftler:innen und internationale Organisationen die Regierungen deshalb immer wieder dazu auf, Stigmatisierung als wichtiges Thema in ihren Strategien und Maßnahmen zur Pandemiebekämpfung zu berücksichtigen [6–8]. Ihre Empfehlungen hoben hervor, dass die Stigmatisierung das mit der Krankheit verbundene Leid auf individueller Ebene dramatisch verstärkt. In einer Ad-hoc-Public-Health-Ethikberatung im Jahr 2020 stellten führende (Medizin‑)Ethiker:innen aus Deutschland zudem heraus, dass eine Vermeidung von Stigmatisierungen nicht nur bei medizinisch vulnerablen Gruppen, also Personen mit erhöhtem Risiko für eine Infektion, nötig sei. Insbesondere auch für Menschen mit sozialen Vulnerabilitäten, die „in Pandemien reproduziert und verstärkt werden“, müssten in der Pandemieplanung „besondere Vorkehrungen hinsichtlich der Erhaltung und Stabilisierung sozialer Gerechtigkeit“ [9] getroffen werden.

Stigmatisierung erzeugt auf individueller Ebene Leid, Exklusion und gesundheitliche Belastungen [4]. Zudem werden möglicherweise Maßnahmen zur Pandemiebekämpfung auf gesellschaftlicher Ebene untergraben [10], weil die Angst vor einer Stigmatisierung die Ablehnung von Behandlungs- und/oder Infektionsschutzmaßnahmen fördert [11, 12]. Angesichts des Eintritts in eine endemische Lage und der Rücknahme aller Infektionsschutzmaßnahmen ist die Stigmatisierung wahrscheinlich kaum noch vorhanden. Dennoch ist es wichtig, Stigmatisierung und ihre Auswirkungen in Deutschland rückblickend systematisch zu beleuchten, um die gewonnenen Erkenntnisse für die Planung von Gesundheitskommunikation in Krisensituationen zukünftig berücksichtigen zu können.

Für Deutschland liegen aktuell nur wenige nicht-repräsentative Studien zu COVID-19-bezogener Stigmatisierung vor [13–15], die alle auf Fremdstigmatisierung ausgerichtet sind. In unserer Studie untersuchen wir hingegen die diesbezügliche „Selbststigmatisierung“, also die Stigmatisierung, die sich ein Individuum im Kontext von COVID-19 selbst auferlegt (siehe auch Ausführungen weiter unten). Ziel ist es dabei zu verstehen, wie die Selbststigmatisierung mit medizinischen und sozialen Vulnerabilitäten zusammenhängt. Zudem betrachten wir, inwiefern Vertrauen in Institutionen Selbststigmatisierung allgemein und bei vulnerablen Gruppen im Besonderen reduzieren kann [16, 17]. Die Datengrundlage ist ein webbasierter Survey (Computer-assisted Web Interview – CAWI), der in einer hinsichtlich zentraler soziodemografischer Merkmale repräsentativen Stichprobe eines Online-Panels durchgeführt wurde (*N* = 2536).

Diese Studie ist die erste, die auf Basis einer repräsentativen Stichprobe COVID-19-bezogene Selbststigmatisierungsprozesse in Deutschland erhebt und damit Erkenntnisse liefern kann, in welchem Ausmaß Selbststigmatisierung im Rahmen der Pandemie vorhanden war und welche Gruppen davon besonders betroffen waren. Diese Ergebnisse sind sowohl für die rückblickende Aufarbeitung der COVID-19-Pandemie als auch für die Entwicklung von Strategien für künftige Krisen der Bevölkerungsgesundheit bedeutsam.

## Hintergründe zur gesundheitsbezogenen Stigmatisierung allgemein und in Bezug auf COVID-19

Stigma ist ein mehrdimensionales soziologisches bzw. sozialpsychologisches Konzept. Die Stigmatisierung beschreibt dabei einen Prozess, bei dem die „Elemente der Etikettierung, Stereotypisierung, Abgrenzung, des Statusverlusts und der Diskriminierung in einer Machtsituation zusammentreffen, die es den Komponenten des Stigmas ermöglicht, sich zu entfalten“ (aus dem Englischen nach [18]). Gesundheitsbezogene Stigmata entfalten sich dabei zunächst über die Kennzeichnung von Individuen oder Gruppen durch negative Attribute. Im Folgenden können sich negative Einstellungen manifestieren, die ablehnendes Verhalten oder auch aktive Diskriminierung hervorrufen [12].

Im Kontext von Infektionskrankheiten sind gesundheitsbezogene Stigmatisierungen vor allem aus der Forschung zum HI-Virus bekannt [19–21]. Dabei verfügen nicht infizierte Personen zwar oft über Wissen zur Gefährlichkeit der Erkrankung, aber nur vereinzelt über Detailwissen, zum Beispiel zu Übertragungswegen. Der Mangel an Informationen kann bei nicht infizierten Personen ein Gefühl der Überforderung auslösen. Um diese Überforderung zu kanalisieren, kann infizierten Personen in einem ersten Schritt ein Selbstverschulden an der Infektion attestiert werden. Erklärungsmuster können dann sein, dass die infizierte Person zu viel Kontakt mit Infizierten hatte oder allgemein zu wenig Eigenverantwortung beim Infektionsschutz übernimmt. Die infizierte Person kann außerdem als gefährlich für die eigene Gesundheit eingestuft, herabgesetzt, gemieden oder sogar aktiv diskriminiert werden.

Hinzu kommt, dass Stigmatisierungsprozesse oftmals nicht nur die Gesundheit und Lebensqualität von stigmatisierten Personen, sondern auch deren Partner:innen, Familien und Freund:innen beeinträchtigen [19, 22]. Aus der Literatur zu Infektionskrankheiten ist zudem bekannt, dass sich negative Einstellungen gegenüber infizierten Personen besonders verstärken, wenn nicht infizierte Personen davon ausgehen, dass sie selbst potenziell gefährdet sind sich anzustecken (siehe z. B. [19, 23, 24]).

Es ist daher zu vermuten, dass auch bei einer Infektion mit SARS-CoV‑2 Stigmatisierungsprozesse auftreten können [4, 25, 26]. In der internationalen Literatur, die im Rahmen der Coronapandemie bisher zu diesem Thema entstanden ist, zeigt sich, dass die mit COVID-19 verbundene Stigmatisierung weit verbreitet, vielfältig und dynamisch ist [27–29]. Durch den Ursprung von COVID-19 in China wurde gerade zu Beginn der Pandemie eine sich schnell entwickelnde Stigmatisierung von Personen asiatischer Abstammung beobachtet [3], die teilweise sogar in physischer Gewalt mündete [30] und psychische Beeinträchtigungen bei dieser Gruppe nach sich zog [31].

In der Folgezeit berichteten insbesondere Beschäftigte im Gesundheits- und Sozialwesen, denen ein höheres Infektionsrisiko zugeschrieben wurde, über Stigmatisierungserfahrungen [28]. Die Stigmatisierung dieser Berufsgruppen war mit einem erhöhten Risiko für Depressionen oder Angststörungen assoziiert und zwar unabhängig davon, ob eine Infektion tatsächlich bestand [32]. Diese Befunde legen nahe, dass medizinische und soziale Vulnerabilitäten mit Stigmatisierungen verbunden sein könnten, da benachteiligten Gruppen ein höheres Infektionsrisiko zugeschrieben wird. Darüber hinaus können bereits bestehende Stereotype gegenüber Minderheiten aktiviert werden. Eine Studie, die diesen Zusammenhang für Deutschland systematisch untersucht, liegt aktuell allerdings noch nicht vor.

Stigmatisierung durch andere kann Diskriminierung unterschiedlichen Ausmaßes (bis hin zu offener Gewalt) beinhalten [18]. Die Selbststigmatisierung ist definiert als eine Form der Stigmatisierung, bei der sich Individuen ein Stigma selbst auferlegen, wenn sie sich einer bestimmten Gruppe zugehörig fühlen, die stigmatisiert wird [33]. Individuen gehen dabei davon aus, dass sie aufgrund ihrer Infektion bzw. ihrer Vulnerabilität für eine Infektion nicht die gleiche Wertschätzung verdienen wie Menschen ohne Infektion oder Erkrankung. Sie fühlen sich dann abgewertet und ausgegrenzt und reagieren auf die erwartete Stigmatisierung mit Scham und Rückzug [15]. Es kann zudem sein, dass sie die Infektion oder Erkrankung verheimlichen, um sich der angenommenen Stigmatisierung nicht aussetzen zu müssen [7, 24].

Selbststigmatisierung ist damit auch gesundheitsrelevant. Denn eine krankheitsbezogene Stigmatisierung kann das Risiko depressiver Symptome, ein niedrigeres Selbstwertgefühl und weitere gesundheitliche Beeinträchtigungen verstärken [32, 34]. Darüber ist eine hohe Selbststigmatisierung und die damit oft verbundene Verheimlichungsstrategie mit einer geringeren Befolgung von Infektionsschutzstrategien (z. B. Testen, Quarantäne) und der Inanspruchnahme von Gesundheitsleistungen assoziiert [11, 12, 20].

Ein zentraler Einflussfaktor zur Senkung der krankheitsbezogenen Stigmatisierung im Allgemeinen und damit auch der Selbststigmatisierung ist Vertrauen [17]. Vertrauen entsteht dann, wenn Individuen positive Reaktionen erfahren, wenn sie ihre Verletzlichkeit gegenüber anderen Personen, Institutionen oder Akteuren offenlegen [35, 36]. So entsteht Vertrauen in einer Ärzt:innen-Patient:innen-Beziehung vor allem, wenn die Patientin oder der Patient ein medizinisches Problem offenlegt und die Ärztin oder der Arzt die eigenen Ressourcen einsetzt, um es zu lösen [37]. Hall et al. [38] zeigen in diesem Kontext, dass Vertrauen von Patient:innen gegenüber dem ärztlichen oder dem Pflegepersonal einer Einrichtung die Wahrscheinlichkeit erhöht, dass dieses Vertrauen auch auf andere Personengruppen im Umfeld der Patient:innen übertragen wird. Stärkeres Vertrauen in staatliche Stellen und Akteure des Gesundheitswesens erhöht gleichsam die Zustimmung zu Richtlinien des öffentlichen Gesundheitswesens, zum Beispiel der Ergreifung von Vorsichtsmaßnahmen gegen Infektionskrankheiten [39] oder der regelmäßigen Untersuchung auf Infektionen [40].

Dieser Beitrag verfolgt daher zwei Ziele. Das erste Ziel ist es, den Zusammenhang zwischen medizinischen und sozialen Vulnerabilitäten auf die COVID-19-bezogene Selbststigmatisierung zu untersuchen. Das zweite Ziel ist es, die Bedeutung des Vertrauens in Institutionen in Hinblick auf Selbststigmatisierung zu erfassen und zu prüfen, inwiefern sich der Einfluss des Vertrauens bei vulnerablen Gruppen unterscheidet. Die Ergebnisse sollen damit einen Überblick über COVID-19-bezogene Selbststigmatisierung und den Einfluss des Vertrauens in Institutionen geben, aus denen sich Hinweise auf Antistigmatisierungsmaßnahmen ableiten lassen.

## Methoden

Die Daten für die Analyse stammen aus einer webbasierten Befragung (CAWI), welche im Rahmen des Nachwuchsforschungsprojektes „Medikalisierung und Psychologisierung sozialer Probleme – Chancen und Herausforderungen für Sozialpolitik“ (MEPYSO) durchgeführt wurde [41]. Der Erhebungszeitraum erstreckte sich von Dezember 2020 bis Januar 2021 und lag damit genau in der zweiten COVID-19-Welle in Deutschland [42]. Mittels eines Quotenstichprobenverfahrens wurde innerhalb des YouGov-Online-Panels für Deutschland eine Stichprobe von 2661 Teilnehmer:innen zur Beantwortung unseres Fragebogens eingeladen. 125 Personen (4,7 %) sagten das Interview vorzeitig ab. Die verbleibende Analysestichprobe von *N* = 2536 ist repräsentativ für die deutsche Erwachsenenbevölkerung in Bezug auf die Schlüsselvariablen Geschlecht, Bildung, Bundesland, Wohnort und Alter.^1^ Der Median der Bearbeitungszeit der Befragung betrug 15 min (x_0,25_ = 8; x_0,75_ = 22). Die Befragten erhielten von YouGov ein Incentive von 500 Token (1 €) und wurden am Ende sorgfältig über den Kontext der Studie aufgeklärt.

Das primäre Interesse dieser Studie liegt im Verständnis von COVID-19-bezogener Selbststigmatisierung. Da keine validierten Skalen zu dieser Thematik vorlagen, haben wir eine eigene Skala bestehend aus 7 Items zur Operationalisierung des Konstruktes entwickelt (Itemformulierungen siehe Tab. 1). Die Befragten konnten ihre Zustimmung auf einer 7‑stufigen Likert-Skala angeben, die von 0 = „stimme überhaupt nicht zu“ bis 6 = „stimme voll und ganz zu“ reichte. Eingeleitet wurde mit der Frage: „Wie würden Sie reagieren, wenn Sie sich selbst mit dem Coronavirus angesteckt hätten?“ Im Rahmen der Regressionsdiagnostik zeigte sich, dass die Bewertungen der Einzelitems in ihren Verteilungen zum Teil stark voneinander abweichen und zumindest nicht ausgeschlossen werden konnte, dass die Residuen heteroskedastisch sind, also in unterschiedliche Richtungen streuen. Zur Konstruktion der Skala wurden die Items daher z‑standardisiert und zu einem Mittelwertindex zusammengefasst. Die interne Konsistenz liegt bei einem Cronbachs Alpha von 0,8. Eine darüber hinaus durchgeführte Faktorenanalyse (Hauptachsenanalyse) bestätigte die 1‑faktorielle Lösung mit einem Kaiser-Meyer-Olkin-Kriterium von 0,82.

**Table 1:** 

Item	Itemformulierung
Verurteilung	Ich würde mir Sorgen machen, dass andere Personen mich verurteilen, wenn ich ihnen von der Infektion erzähle
Verheimlichung	Ich würde nur bestimmten Leuten von einer Infektion erzählen und diese bitten, es für sich zu behalten
Wachsamkeit	Ich würde mich schuldig fühlen, weil ich nicht wachsam genug war
Vermeidung	Ich hätte Angst, dass mich Personen auch nach überstandener Infektion erstmal meiden
Quarantäne	Ich hätte Angst, dass es mir andere Personen übelnehmen, wenn sie wegen mir in Quarantäne müssen
Unsicherheit	Ich würde mir große Sorgen machen und mich unsicher fühlen
Infektion	Ich hätte Angst, andere Personen anzustecken

Die Antwort erfolgte auf einer 7‑stufigen Likert-Skala von 0 = „stimme überhaupt nicht zu“ bis 6 = „stimme voll und ganz zu“. *Quelle*: Corona-Studie des Projektes MEPYSO [41]

Für die Messung der medizinischen Vulnerabilität baten wir die Befragten anzugeben, ob sie gemäß der Definition vom Robert Koch-Institut (RKI) den Risikogruppen für einen schweren Verlauf von COVID-19 angehören (Nein/Ja).^2^ Zudem sollten die Befragten einschätzen, für wie wahrscheinlich sie es halten, dass a) sie sich selbst und b) nahe Angehörige sich innerhalb der nächsten zwei Monate infizieren (7-stufige Likert-Skala von 1 = „gering“ bis 7 = „hoch“). Weiterhin baten wir um die subjektive Einschätzung des eigenen Gesundheitszustandes (5-stufige Likert-Skala von 1 = „sehr schlecht“ bis 5 = „sehr gut“). Aufgrund des mit dem Alter steigenden Risikos eines schweren Verlaufs zählen wir das Alter ebenfalls zu den medizinisch relevanten Größen. Wir unterscheiden im Folgenden zwischen den Altersgruppen 18–35, 36–45, 46–65 und ab 66 Jahre.

Die soziale Vulnerabilität bilden wir über 7 soziodemografische Variablen ab: Geschlecht (weiblich, männlich), Bildungsniveau (niedrig: höchstens Hauptschulabschluss, mittel: höchstens Realschulabschluss, hoch: mindestens Fachhochschulreife), Haushaltseinkommen (weniger als 1000 €, 1000–1999 €, 2000–2999 €, 3000 € und mehr), Erwerbsstatus (erwerbstätig, Rente, Bezug von Transferleistungen und andere Erwerbsformen), Staatsbürgerschaft (deutsche/ausländische oder ausländische und deutsche), Haushaltsgröße und Vorhandensein minderjähriger Kinder im Haushalt (Nein/Ja).

Zur Messung des Vertrauens in Institutionen haben wir uns an etablierte Vertrauensskalen (European Social Survey, GESIS Online Panel) angelehnt und diese um weitere Akteure, die für die Pandemiebekämpfung wichtig waren, ergänzt. Die Befragten konnten angeben, inwieweit sie verschiedenen Professionen (z. B. Virolog:innen) und Institutionen (z. B. Weltgesundheitsorganisation – WHO) im Umgang mit der Coronapandemie vertrauen. Das jeweilige Maß an Vertrauen konnte auf einer 10-stufigen Likert-Skala angegeben werden, deren Endpunkte mit 0 „vertraue überhaupt nicht“ und 10 „vertraue voll und ganz“ beschriftet waren. Zur Erhöhung der internen Konsistenz wurde das Vertrauen in die Hausärzt:in, Lehrer:innen, Arbeitgeber:in und das Krankenpflegepersonal nicht berücksichtigt. Für die Analyse verwenden wir den Mittelwertindex aller anderen Einzelitems (Cronbachs Alpha = 0,97).^3^

Die Item-Nonresponse reicht für einzelne Variablen von (fast) keiner bis zu 18,61 % für das Haushaltseinkommen.^4^ Auch wenn die Zahl der fehlenden Werte für einzelne Variablen moderat ist, führt das Verfahren der *Listwise Deletion* dazu, dass insgesamt eine große Zahl von Fällen ausgeschlossen wird. Die Entscheidung, diese Fälle nicht zu berücksichtigen, hätte zu ineffizienten und inkonsistenten Schätzungen führen können [43]. Daher wurden die OLS-Regressionsmodelle (engl. Ordinary Least Squares) mit Multiple Imputed Chain Equation (MICE; [44]), einschließlich Survey-Gewichte, und den mi-Befehlen in STATA 17 geschätzt. Tab. 2 präsentiert die gewichteten Mittelwerte bzw. relativen Häufigkeiten sowie die absoluten Häufigkeiten der im Modell verwendeten Variablen nach *N* = 20 Imputationen.

**Table 2:** 

	Deskriptive Statistiken	
	Absolute Häufigkeit	Relative Häufigkeit oder *Mittelwert*
***Soziale Vulnerabilität***		
**Geschlecht**		
Männlich	1238	0,49
Weiblich	1298	0,51
**Staatsbürgerschaft**		
Deutsch	2384	0,94
Andere (und deutsch)	152	0,06
**Bildungsniveau**		
Niedrig	557	0,22
Mittel	1288	0,51
Hoch	691	0,27
**Haushaltseinkommen**		
Weniger als 1000 €	310	0,12
1000–1999 €	736	0,29
2000–2999 €	633	0,25
3000 € und mehr	857	0,34
**Erwerbsstatus**		
Erwerbstätig	1381	0,54
Rente	786	0,31
Transferleistungen	158	0,06
Andere	211	0,08
**Haushaltsgröße**	2536	*2,22*
**Kinder im Haushalt**		
Nein	1944	0,77
Ja	592	0,23
***Medizinische Vulnerabilität***		
**Risikogruppe für einen schweren Verlauf von COVID-19**		
Nein	1034	0,41
Ja	1502	0,59
**Eigene Infektionswahrscheinlichkeit**	2536	*2,12*
**Infektionswahrscheinlichkeit Umfeld**	2536	*2,57*
**Allg. Gesundheitszustand**		
(Sehr) schlecht	321	0,13
Mittelmäßig	900	0,35
(Sehr) gut	1315	0,52
**Alter in Jahren**		
18–35	623	0,25
36–45	378	0,15
46–55	547	0,22
56–65	406	0,16
Ab 66	582	0,23
**Vertrauen in Institutionen (Mittelwertindex)**	2536	*6,09*
**Selbststigmatisierung (Mittelwertindex)**	2536	*−0,01*

*Quelle*: Corona-Studie des Projektes MEPYSO [41], eigene Berechnungen

## Ergebnisse

**Figure 1:**
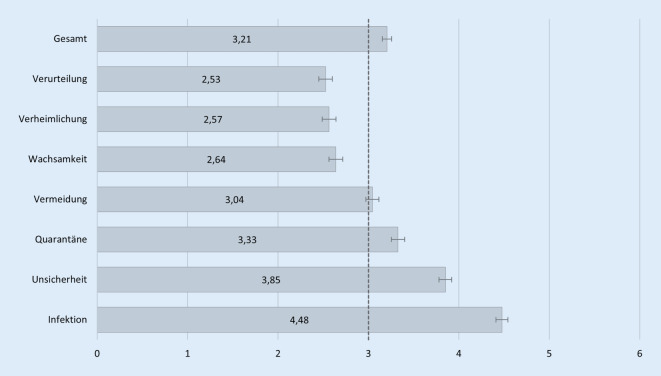

Insgesamt liegt die Selbststigmatisierung mit 3,21 (95 %-KI: 3,15–3,26) leicht über dem Skalenmittel von 3 (Abb. 1). Es kann also davon ausgegangen werden, dass zumindest zum Zeitpunkt der Erhebung eine Infektion mit COVID-19 zum Teil mit Selbststigmatisierung einherging. Die durchschnittliche Zustimmung zu den einzelnen Selbststigmatisierungsitems schwankt zwischen 2,53 (95 %-KI: 2,45–2,60) für die Angst vor Verurteilung durch andere bis hin zu 4,48 (95 %-KI: 4,41–4,54) für die Furcht, andere Personen anzustecken. Hohen Zuspruch erhielt ebenfalls die Aussage, dass mit einer Infektion ein Gefühl von großer Unsicherheit einhergeht. Aber auch die Sorge davor, dass Personen es einem übel nehmen könnten, wenn diese in Quarantäne müssen, ist überdurchschnittlich hoch. Der eigenen Verurteilung aufgrund mangelnder Wachsamkeit sowie der Absicht, die Infektion zu verheimlichen, wurden hingegen weniger stark zugestimmt.

Im Rahmen von gewichteten multiplen OLS-Regressionen wurde geprüft, inwiefern soziale Vulnerabilität, medizinische Vulnerabilität und Vertrauen in Institutionen mit der Selbststigmatisierung assoziiert sind. Tab. 3 zeigt 4 Regressionsmodelle, in denen schrittweise Variablenblöcke ergänzt werden. In Modell 1 wurde zunächst der Zusammenhang mit den Indikatoren der sozialen Vulnerabilität geschätzt. Dieser Variablenblock erklärt 2 % der Gesamtvarianz. Frauen weisen dabei eine signifikant höhere Selbststigmatisierung als Männer auf. Auch Personen mit ausländischer bzw. doppelter Staatsbürgerschaft weisen im Vergleich mit Personen mit einem deutschen Pass einen signifikant höheren Wert auf der Skala der Selbststigmatisierung auf. In Hinblick auf das Einkommen zeigen alle niedrigeren Gruppen im Vergleich zur Referenzkategorie der höchsten Einkommensgruppe (3000 € oder mehr) eine erhöhte Selbststigmatisierung auf. Statistisch signifikant ist der Unterschied allerdings nur für die Gruppe der Personen, die 2000 € bis unter 3000 € verdienen. Rentner haben im Vergleich zu erwerbstätigen Personen eine signifikant höhere Selbststigmatisierung angegeben. Keine signifikanten Effekte ergeben sich beim Bildungsniveau, der Haushaltsgröße und bei Kindern im Haushalt.

**Table 3:** 

*Abhängige Variable: Selbststigmatisierung*	Modell 1		Modell 2		Modell 3		Modell 4	
*Unabhängige Variablen*	Koef.	S. E.	Koef.	S. E.	Koef.	S. E.	Koef.	S. E.
**Soziale Vulnerabilität**								
Geschlecht (Ref.: männlich)								
*Weiblich*	0,117***	0,030	0,115***	0,029	0,099***	0,028	0,192***	0,049
Staatsbürgerschaft (Ref.: deutsch)								
*Andere (und deutsch)*	0,132*	0,065	0,110	0,059	0,102	0,057	0,097	0,057
Bildungsniveau (Ref.: hoch)								
*Niedrig*	0,017	0,048	0,025	0,046	0,029	0,045	0,034	0,045
*Mittel*	0,034	0,035	0,036	0,033	0,047	0,033	0,050	0,033
Haushaltseinkommen (Ref.: 3000 € u. mehr)								
*Weniger als 1000* *€*	0,019	0,062	0,026	0,061	0,047	0,059	0,051	0,059
*1000–1999* *€*	0,086	0,049	0,081	0,046	0,090*	0,045	0,089	0,045
2000–2999 €	0,086*	0,042	0,060	0,040	0,048	0,039	0,047	0,039
Erwerbsstatus (Ref.: erwerbstätig)								
*Rente*	0,102**	0,036	0,023	0,055	0,014	0,053	0,012	0,053
*Transferleistungen*	−0,068	0,067	−0,092	0,066	−0,089	0,063	−0,090	0,063
*Andere*	0,051	0,068	0,091	0,068	0,078	0,067	0,078	0,066
Haushaltsgröße	0,027	0,017	0,026	0,016	0,028	0,016	0,026	0,016
Kinder im Haushalt (Ref.: Nein)								
*Ja*	−0,022	0,046	−0,064	0,045	−0,062	0,045	−0,060	0,045
**Medizinische Vulnerabilität**								
Risikogruppe für SARS-CoV‑2 (Ref.: Nein)								
*Ja*	–	–	0,074	0,040	0,065	0,038	0,144**	0,051
Eigene Infektionswahrscheinlichkeit	–	–	0,112***	0,017	0,097***	0,017	0,096***	0,017
Infektionswahrscheinlichkeit Umfeld	–	–	0,051***	0,015	0,027	0,014	0,027	0,014
Allg. Gesundheitszustand (Ref.: (sehr) gut)								
*(Sehr) schlecht*	–	–	0,182***	0,049	0,202***	0,049	0,200***	0,049
*Mittelmäßig*	–	–	0,095**	0,033	0,105**	0,032	0,106***	0,032
Alter in Jahren (Ref.: 46–55)								
*18–35*	–	–	0,017	0,047	0,017	0,046	0,018	0,045
*36–45*	–	–	0,033	0,051	0,076	0,050	0,077	0,050
*56–65*	–	–	−0,053	0,048	−0,052	0,047	−0,051	0,047
*Ab 66*	–	–	0,061	0,059	0,031	0,058	0,036	0,057
**Vertrauen**								
Vertrauen in Institutionen	–	–	–	–	0,162***	0,024	0,163***	0,024
Quadriertes Vertrauen in Institutionen	–	–	–	–	−0,010***	0,002	−0,010***	0,002
**Interaktionseffekt**								
Interaktion Risikogruppe und Geschlecht	–	–	–	–	–	–	−0,157*	0,062
**Adj. R**^**2**^	0,02		0,11		0,16		0,17	
***N***	2536		2536		2536		2536	

*Quelle*: Corona-Studie des Projektes MEPYSO [41], eigene Berechnungen

*Koef.* Regressionskoeffizient, *Ref*. Referenzgruppe, *S. E.* Standardfehler, * *p* < 0,05, ** *p* < 0,01, *** *p* < 0,001

In Modell 2 werden nun zusätzlich die Variablen zur medizinischen Vulnerabilität berücksichtigt, wodurch weitere 9 % der Gesamtvarianz erklärt werden können. Es ist ersichtlich, dass verschiedene Indikatoren der medizinischen Vulnerabilität mit Selbststigmatisierung assoziiert sind. Während das Alter und die Zugehörigkeit zur Risikogruppe nicht signifikant assoziiert sind, gehen ein höheres selbsteingeschätztes Infektionsrisiko, ein höheres Infektionsrisiko des sozialen Umfelds sowie ein (sehr) schlechter oder mittelmäßiger Gesundheitszustand mit einer höheren Selbststigmatisierung einher. Mit Hinzunahme der medizinischen Vulnerabilität verlieren die soziodemografischen Effekte teilweise an Bedeutung und nur der Geschlechtereffekt ist weiterhin signifikant.

**Figure 2:**
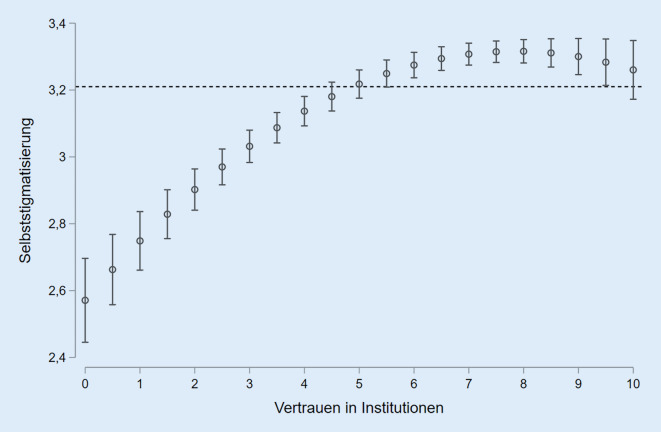

Modell 3 berücksichtigt darüber hinaus das Vertrauen in Institutionen, womit 5 % der Gesamtvarianz erklärt werden können. Hier zeigte die Regressionsdiagnostik, dass ein kurvilinearer Zusammenhang vorliegt. Das heißt, mit zunehmendem Vertrauen steigt die Selbststigmatisierung zunächst an, bis sich dieser Effekt abschwächt und die Selbststigmatisierung bei sehr hohen Vertrauenswerten wieder leicht sinkt (Abb. 2). Aus diesem Grund wurde zusätzlich der quadratische Term der Vertrauensvariable aufgenommen. Durch die Berücksichtigung des Vertrauens verringert sich der Koeffizient des Infektionsrisikos des Umfeldes und ist nicht länger verallgemeinerbar. Der Unterschied zwischen der zweitniedrigsten und der höchsten Einkommensgruppe wird hingegen knapp signifikant.

**Figure 3:**
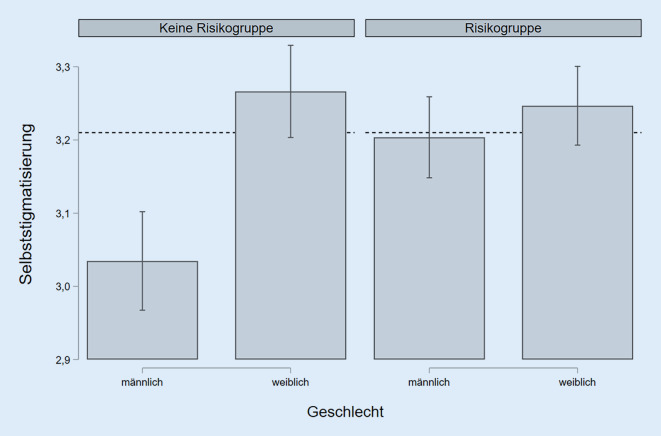

Das finale Modell 4 wird ergänzt durch einen Interaktionseffekt der Geschlechter- und Risikogruppenvariable. Dieser trägt mit 1 % zur Varianzaufklärung bei, ist signifikant und führt außerdem dazu, dass der Effekt der Risikogruppenzugehörigkeit es ebenfalls wird. Wie in Abb. 3 dargestellt, ergibt sich nun folgender Zusammenhang zwischen Geschlecht und Risikogruppenzugehörigkeit in Bezug auf die Selbststigmatisierung: Frauen, die nicht der Risikogruppe angehören, haben eine höhere Selbststigmatisierung als Männer. Gehören Frauen zur Risikogruppe, verbleibt die Höhe der Stigmatisierung aber auf einem in etwa gleichen Niveau. Bei den Männern ergibt sich hingegen ein deutlich höheres Niveau an Selbststigmatisierung, wenn sie zur Risikogruppe gehören. Das bedeutet, dass sich die Geschlechter nur signifikant hinsichtlich ihrer Selbststigmatisierung unterscheiden, wenn sie nicht einer der Risikogruppen für einen schweren Verlauf von COVID-19 angehören. Die Effekte der anderen Modellvariablen verändern sich hierdurch allenfalls marginal. Es gab keine weiteren signifikanten Interaktionen mit Variablen der medizinischen und sozialen Vulnerabilität.

## Diskussion

In dieser Studie haben wir auf Basis einer repräsentativen Online-Befragung, die während der 2. Corona-Welle durchgeführt wurde, untersucht, wie medizinische und soziale Vulnerabilität sowie das Vertrauen in Institutionen mit COVID-19-bezogener Selbststigmatisierung assoziiert sind. Die Ergebnisse zeigen, dass die Befragten im Durchschnitt eine Selbststigmatisierung angaben, die leicht über dem Skalenmittelwert liegt. Auch wenn Befragte vermehrt Angst davor hatten, andere anzustecken, und sich unsicher fühlten, gab es wenig Zustimmung zu Verheimlichungs‑, Verurteilungs- und Vermeidungsstrategien. Die Selbststigmatisierung ist damit selbst in einer Hochphase der Pandemie insgesamt moderat. Außerdem ergab sich, dass sozial vulnerable Gruppen (hinsichtlich Bildung, Einkommen, Erwerbsstatus, Staatsbürgerschaft, Haushaltsgröße und Kinder im Haushalt) nicht signifikant stärker zu Selbststigmatisierung tendieren mit der Ausnahme von Frauen. Frauen wiesen generell eine stärkere Tendenz zur Selbststigmatisierung als Männer auf, allerdings nur, wenn diese nicht zur Risikogruppe für COVID-19 zählten. Personen, die eine medizinische Vulnerabilität aufwiesen, d. h. sich selbst als infektionsgefährdet ansahen oder einen schlechten Gesundheitszustand angaben, neigten jedoch stärker zu Selbststigmatisierung als nicht vulnerable Personen. Entgegen den theoretischen Erwartungen war ein höheres Vertrauen (bis zu einem gewissen Punkt) mit einer Tendenz zur Selbststigmatisierung assoziiert.

Die Studie weist mehrere Limitationen auf. Bei den verwendeten Daten handelt es sich um eine nicht-experimentelle Studie im Querschnittsdesign. Daher sind keine kausalen Schlussfolgerungen möglich. Die Skala zur Selbststigmatisierung wurde aufgrund der begrenzten Zeit für die Durchführung der Studie zu einem frühen Zeitpunkt in der Pandemie nur in kleinem Umfang pre-getestet; eine größere Validierungsstudie liegt noch nicht vor. Da es sich um Selbsteinschätzungen handelt, besteht auch das Risiko für sozial erwünschte Antworten. Aufgrund der Durchführung als Online-Studie ist die soziale Erwünschtheit jedoch als weniger bedeutsam einzuschätzen als in anderen Erhebungsformen. Darüber hinaus sind die Einschätzungen zur Selbststigmatisierung und zu den medizinischen Vulnerabilitäten im zeitlichen Kontext zu interpretieren. Auch wenn das grundsätzlich gültig ist, ist dieser Aspekt aufgrund der hohen Dynamik der Pandemie, des Wissens und der geltenden Maßnahmen als besonders wichtig einzuschätzen. Im Erhebungszeitraum von Dezember 2020 bis Januar 2021 waren primär noch SARS-CoV-2-Viren des Wildtypus dominant, Deutschland befand sich in einer Phase des Lockdowns und der Großteil der Bevölkerung war noch ungeimpft, da erst im Dezember 2020 die ersten Impfangebote gestartet hatten. Die Ergebnisse zur Selbststigmatisierung sind damit nicht einfach auf spätere Pandemiephasen oder die aktuelle Situation übertragbar.

Trotz dieser Limitationen weist die Studie auch wichtige Stärken auf. Die vorliegende Arbeit ist die erste, die Selbststigmatisierung im Kontext von COVID-19 in Deutschland untersucht. Außerdem basiert sie auf einer Stichprobe, die hinsichtlich zentraler Merkmale bevölkerungsrepräsentativ ist und damit den Kenntnisstand erweitert, der bisher auf eingeschränkten Stichproben oder Convenience Sampling beruhte [13–15]. Ein direkter Vergleich mit Studien, die Fremdstigmatisierung adressieren oder Ergebnisse aus anderen Ländern berichten, ist schwierig. Dennoch stimmen unsere Ergebnisse mit den Befunden einer Studie mit Beschäftigten im Gesundheitssektor in 173 Ländern überein, die dokumentiert, dass Stigmatisierung in Europa deutlich seltener berichtet wurde als in Afrika, Asien, Nord- und Lateinamerika. Ebenso berichten Dye et al. [28] aus einer Studie mit COVID-19-Genesenen aus Deutschland ein moderates Ausmaß an Stigmatisierung. Kulturelle Faktoren könnten hier demnach eine Rolle spielen.

Unser Beitrag zeigt auch, dass Selbststigmatisierung in dieser Phase der Pandemie außer bei Frauen keine zusätzliche Belastung für sozial vulnerable Gruppen dargestellt hat. Bisherige Studien kamen hier zu unterschiedlichen Ergebnissen [14, 15], beruhen allerdings auch auf abweichenden Stichproben (junge Menschen, COVID-19-Genesene). Die stärkere Selbststigmatisierung bei Frauen, die sich aus unseren Daten ergibt, wurde aus anderen COVID-19-bezogenen Studien bisher nicht berichtet. Eine Erklärung wäre, dass Selbststigmatisierung mit psychischer Belastung assoziiert ist, die bei Frauen in der Pandemie ausgeprägter war [45–47], u. a. wegen stark zunehmender Care-Aufgaben (z. B. Kinderbetreuung, Angehörigenpflege) und psychischer Belastung (Mental Load). Darüber hinaus ist aus der Literatur ein stabiler Geschlechtereffekt bei der Einschätzung von Risiken bekannt, d. h., dass Männer Risiken grundsätzlich geringer einschätzen als Frauen [48]. Da das eingeschätzte Risiko relevant für die Selbststigmatisierung ist, könnte dieser Mechanismus unseren Interaktionseffekt erklären: Ohne weitere Informationen bzw. Hinweise auf ein persönliches Risiko schätzen Männer ihr Risiko geringer ein. Liegen begründete Hinweise auf ein höheres Risiko vor, sind die grundsätzlichen Unterschiede in der Einschätzung zwischen den Geschlechtern für die Selbststigmatisierung nicht mehr relevant.

## Fazit

Gerade im ersten Jahr der Pandemie waren die Identifikation und wiederholte Benennung von Risikogruppen ein zentraler Bestandteil der öffentlichen Kommunikation. Die Unterteilung der Bevölkerung in Gruppen mit höheren und geringeren Risiken könnte dabei zu einer stärkeren Selbststigmatisierung dieser Gruppen beigetragen haben. Unsere Studie zeigt, dass COVID-19-bezogene Selbststigmatisierung nur moderat ausgeprägt war und sozial vulnerable Gruppen nicht signifikant häufiger betroffen sind. Jedoch tendierten Personen mit einer höheren medizinischen Vulnerabilität stärker dazu, sich selbst zu stigmatisieren.

In ihren Empfehlungen zur Vermeidung von COVID-19-bezogenen Stigmatisierungsprozessen weist die WHO darauf hin, dass Sprache hier eine wichtige Rolle spielt. Beispielsweise ist die Formulierung „Personen, die ein höheres Risiko aufweisen“, der Bezeichnung „Risikogruppen“ vorzuziehen. Die starke Betonung von Risiken und der Bedrohung durch die Pandemie kann im Vergleich zur Kommunikation der Bedeutung von Maßnahmen Stigmatisierungsprozesse verstärken. Dass Kommunikationsmaßnahmen einen Einfluss auf Selbststigmatisierung haben könnten, deutet sich auch im Zusammenhang mit dem Vertrauen in Institutionen an. Denn unsere Ergebnisse weisen darauf hin, dass Personen mit einem hohen Vertrauen in Institutionen eher zu Selbststigmatisierung neigen. Kommunikation von öffentlichen Institutionen, die auf Warnungen zur Wachsamkeit und Betonung individueller Verantwortung ausgerichtet ist, wird von Personen mit einem höheren Vertrauen in die kommunizierenden Institutionen stärker internalisiert und kann zu Selbststigmatisierung führen. Risikokommunikation sollte daher zukünftig die Auswirkungen auf Stigmatisierungsprozesse als wichtiges Kriterium in der Kommunikationsstrategie berücksichtigen. Dazu wäre es wichtig, Daten zu Stigmatisierung im Verlauf von Pandemien regelmäßig zu erheben, um Stigmatisierungstendenzen und -trends zu erfassen, um dann gezielt entgegenwirken zu können.

## Supplementary Information





## Footnotes

1 Für einen Vergleich der Stichprobendaten mit den Zensus-Daten siehe Tab. Z1 im Onlinematerial.

2 Vorgegebene Risikogruppen: ältere Personen (mit stetig steigendem Risiko für einen schweren Verlauf ab etwa 50–60 Jahren); Raucher; sehr stark übergewichtige Menschen; Personen mit bestimmten Vorerkrankungen (ohne Rangfolge): des Herz-Kreislauf-Systems (z. B. koronare Herzerkrankung und Bluthochdruck), chronische Lungenerkrankungen (z. B. COPD), chronische Nieren- und Lebererkrankungen, Patienten mit Diabetes mellitus (Zuckerkrankheit), Patienten mit einer Krebserkrankung, Patienten mit geschwächtem Immunsystem (z. B. aufgrund einer Erkrankung, die mit einer Immunschwäche einhergeht oder durch die regelmäßige Einnahme von Medikamenten, die die Immunabwehr beeinflussen und herabsetzen können, wie z. B. Cortison).

3 Die Mittelwerte zum Vertrauen in die einzelnen Personengruppen und Institutionen, aus denen sich der Index zusammensetzt, können in Abb. Z1 im Onlinematerial eingesehen werden.

4 Für eine Übersicht siehe Tab. Z2 im Onlinematerial.
