# Supplementary material for: COVID-19-related self-stigma: association with vulnerability and trust in institutions
Source: Bundesgesundheitsblatt Gesundheitsforschung Gesundheitsschutz. 2023 Jul 3;66(8):835–45. [Article in German] doi: 10.1007/s00103-023-03742-z (PMC10372126; doi:10.1007/s00103-023-03742-z)
Supplement: Supplementary file 1 [file 103_2023_3742_MOESM1_ESM.pdf]

**Tab. Z1: Deskriptive Statistiken der quotenbasierte Befragungsmerkmale (gewichtete Stichprobe, N=2.536) im Vergleich zu Zensusdaten (Alter 18+) aus 2011 ($N_{Zensus}$ = 67.065.739).**

| | Zensus 2011[a] | Stichprobe[b] | |
|---|---|---|---|
| *Kategoriale Variablen* | % | Absolut | % |
| Geschlecht | | | |
| Männlich | 48,80 | 1237 | 48,78 |
| Weiblich | 51,20 | 1299 | 51,22 |
| Bildungsniveau | | | |
| Primäre | 5,00 | 118 | 4,69 |
| Sekundär | 79,40 | 1949 | 77,43 |
| Tertiär | 15,60 | 450 | 17,88 |
| Bundesland | | | |
| Schleswig-Holstein | 3,49 | 84 | 3,31 |
| Hamburg | 2,13 | 60 | 2,37 |
| Niedersachsen | 9,70 | 244 | 9,62 |
| Bremen | 0,81 | 17 | 0,67 |
| Nordrhein-Westphalen | 21,86 | 554 | 21,85 |
| Hessen | 7,44 | 200 | 7,89 |
| Rheinland-Pfalz | 4,97 | 121 | 4,77 |
| Baden-Württemberg | 13,07 | 316 | 12,46 |
| Bayern | 15,45 | 378 | 14,91 |
| Saarland | 1,25 | 28 | 1,10 |
| Berlin | 4,10 | 113 | 4,46 |
| Brandenburg | 3,06 | 87 | 3,43 |
| Mecklenburg-Vorpommern | 2,01 | 52 | 2,05 |
| Sachsen | 5,06 | 144 | 5,68 |
| Sachsen-Anhalt | 2,85 | 68 | 2,68 |
| Thüringen | 2,73 | 70 | 2,76 |
| *Metrische Variablen* | Mittelwert | Mittelwert | Standardabweichung |
| Alter (18+) | 48,99 | 50,71 | 16,22 |

Quellen: [a] Zensus Deutschland 2011 (https://ergebnisse.zensus2011.de) [b] Corona-Studie des Projektes MEPYSO (https://doi.org/10.7802/2465), eigene Berechnungen.

**Tab. Z2: Fehlende Werte („Missings”) vor und nach der Imputation, gewichtete Stichprobe**

| | Vor Multipler Imputation (MI) | | | | | Nach MI (*N* = 2,536) | |
|---|---|---|---|---|---|---|---|
| **Variablen** | *N* | Mis-sing | Mis-sing | M | SE | M | SE |
| Geschlecht | 2536 | 0 | 0 | 0,512 | 0,010 | 0,512 | 0,010 |
| Staatsbürgerschaft | 2438 | 98 | 0,04 | 0,058 | 0,005 | 0,060 | 0,005 |
| Bildungsniveau | 2536 | 0 | 0 | 2,053 | 0,014 | 2,053 | 0,014 |
| Haushaltseinkommen | 2064 | 472 | 18,61 | 2,796 | 0,023 | 2,803 | 0,023 |
| Erwerbsstatus | 2420 | 116 | 4,57 | 0,676 | 0,019 | 0,684 | 0,020 |
| Haushaltsgröße | 2534 | 2 | <0,01 | 2,215 | 0,024 | 2,216 | 0,024 |
| Kinder im Haushalt | 2533 | 3 | <0,01 | 0,233 | 0,009 | 0,233 | 0,009 |
| Risikogruppe für SARS-CoV-2 | 2321 | 215 | 8,48 | 0,598 | 0,010 | 0,592 | 0,010 |
| Eigene Infektionswahrschein-lichkeit | 2156 | 380 | 14,98 | 2,109 | 0,027 | 2,121 | 0,026 |
| Infektionswahrscheinlichkeit | 2181 | 355 | 14,00 | 2,584 | 0,029 | 2,569 | 0,029 |
| Allg. Gesundheitszustand | 2443 | 39 | 3,67 | 1,389 | 0,014 | 1,392 | 0,014 |
| Alter in Jahren | 2536 | 0 | 0 | 1,979 | 0,030 | 1,979 | 0,030 |
| Vertrauen in Institutionen | 2263 | 273 | 10,76 | 6,114 | 0,055 | 6,086 | 0,055 |
| Selbststigmatisierungsindex | 2077 | 459 | 18,10 | 0,000 | 0,015 | -0,009 | 0,015 |

Quelle: Corona-Studie des Projektes MEPYSO (https://doi.org/10.7802/2465), eigene Berechnungen. M=Mittelwert, SE=Standardfehler; M und SE repräsentieren aggregierte Werte auf der Basis von 20 Imputationen.

**Abb. Z1: Mittelwerte des Vertrauens in einzelne Institutionen, gewichtete Stichprobe, N=2.536**

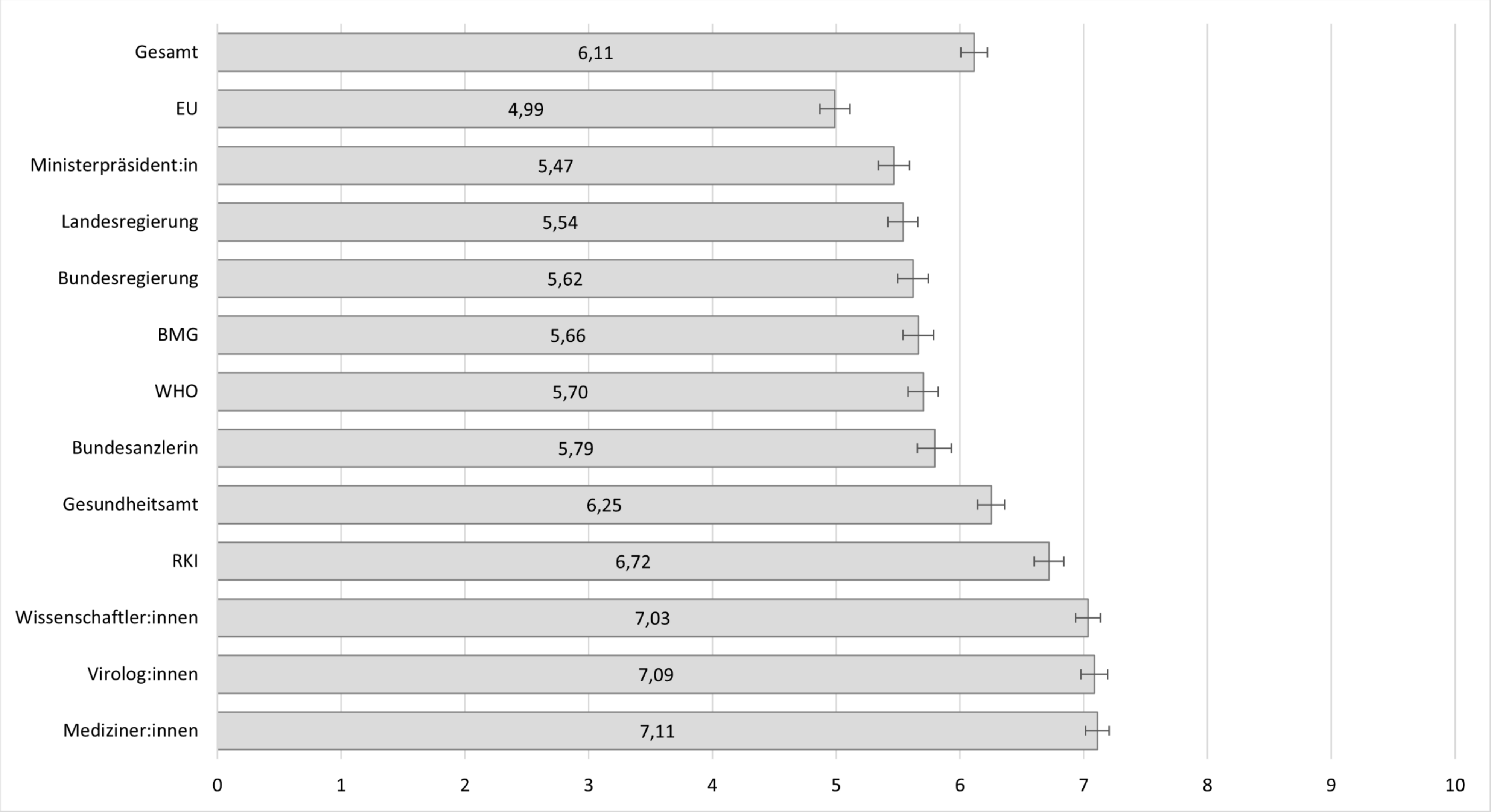


Quelle: Corona-Studie des Projektes MEPYSO (https://doi.org/10.7802/2465), eigene Berechnungen.
